# Low-Temperature Cu/SiO_2_ Hybrid Bonding with Low Contact Resistance Using (111)-Oriented Cu Surfaces

**DOI:** 10.3390/ma15051888

**Published:** 2022-03-03

**Authors:** Jia-Juen Ong, Wei-Lan Chiu, Ou-Hsiang Lee, Chia-Wen Chiang, Hsiang-Hung Chang, Chin-Hung Wang, Kai-Cheng Shie, Shih-Chi Yang, Dinh-Phuc Tran, King-Ning Tu, Chih Chen

**Affiliations:** 1 Department of Materials Science and Engineering, National Yang Ming Chiao Tung University, Hsinchu 30010, Taiwan; jason961213.en08@nycu.edu.tw (J.-J.O.); alex911666.mse06g@g2.nctu.edu.tw (K.-C.S.); nanotwinnedcu.en10@nycu.edu.tw (S.-C.Y.); trandinhphuc1508@gmail.com (D.-P.T.); 2 Department of Materials Science and Engineering, National Chiao Tung University, Hsinchu 30010, Taiwan; 3 Electronic and Optoelectronic System Research Laboratories, Industrial Technology Research Institute (ITRI), Hsinchu 30010, Taiwan; arthurchiu@itri.org.tw (W.-L.C.); joylee@itri.org.tw (O.-H.L.); cwchiang@itri.org.tw (C.-W.C.); mikechang@itri.org.tw (H.-H.C.); jerry_wang@itri.org.tw (C.-H.W.); 4 Department of Materials Science and Engineering, City University of Hong Kong, Hong Kong, China; kntu@cityu.edu.hk; 5 Department of Electrical Engineering, City University of Hong Kong, Hong Kong, China

**Keywords:** Cu/SiO_2_ hybrid bonding, highly (111)-nanotwinned Cu, low temperature bonding, microelectronic packaging

## Abstract

We adopted (111)-oriented Cu with high surface diffusivity to achieve low-temperature and low-pressure Cu/SiO_2_ hybrid bonding. Electroplating was employed to fabricate arrays of Cu vias with 78% (111) surface grains. The bonding temperature can be lowered to 200 °C, and the pressure is as low as 1.06 MPa. The bonding process can be accomplished by a 12-inch wafer-to-wafer scheme. The measured specific contact resistance is 1.2 × 10^−9^ Ω·cm^2^, which is the lowest value reported in related literature for Cu-Cu joints bonded below 300 °C. The joints possess excellent thermal stability up to 375 °C. The bonding mechanism is also presented to provide more understanding on hybrid bonding.

## 1. Introduction

As the evolution of AI and high-performance computing (HPC) devices needs more input and output (I/O) numbers, fine-pitch packaging techniques such as Cu-Cu bonding [1,2,3] or Cu/dielectric hybrid bonding is needed urgently. In addition, 3D IC packaging is at the core of development for advanced package technology to provide high bandwidth and low power consumption. Nowadays, flip-chip solder micro-joints are commonly used for vertical interconnects. However, the size of micro-joints cannot be scaled down to 10 μm due to side wetting of solders and bridging failures.

Cu/oxide hybrid bonding, on the other hand, with oxide or SiCN dielectrics has been adopted for fine-pitch packaging and can be scaled down continuously below the submicron scale [4,5,6,7,8]. Furthermore, copper shows low electrical resistivity (ρ) of 1.7 × 10^−6^ Ω·cm^2^ which is a constant that would be varied by different materials. Thus, it would affect the resistance with the cross-sectional area of the materials that current flows through. On the other hand, silicon oxide has been an optimal dielectric for copper hybrid bonding due to its low coefficient of thermal expansion (CTE) and high bonding energy. However, the current bonding temperature is around 300 °C, which is too high for high-bandwidth memories. Therefore, to reduce thermal budget and thermal stress and to increase alignment accuracy, it is necessary to lower bonding temperature. 

Highly <111>-oriented nanotwinned (nt) Cu possesses low resistivity, low oxidation rate, and high electromigration resistance [9,10,11,12]. More importantly, the (111) surface has the highest diffusivity among all the crystallographic planes in face-centered cubic (FCC) crystals, this is due to the fact that the (111) plane is the most densely packed surface which contributes to high atomic diffusivity [13,14]. The bonding mechanism is attributed to surface creep, which is atomic diffusion under stress gradient at elevated temperatures [15]. Thus, direct bonding can be completed at low temperatures. The size distribution of voids and evolution due to the ripening effect during bonding have also been reported [16]. With the advantage of highly <111> nt-Cu, one can further lower the bonding temperature to 200 °C [17]. However, there is no study reporting the electrical characteristics of the nt-Cu/SiO_2_ hybrid bonds.

In this paper, we adopt the highly <111>-nanotwinned Cu (nt-Cu) and SiO_2_ dielectrics to lower the bonding temperature and pressure. Kelvin and cross-bar structures were designed to measure the electrical resistance and specific contact resistance. The results show that excellent bonding interfaces can be obtained at 200 °C under 1.06 MPa. The electrical resistance is quite stable from 25 °C to 375 °C. Furthermore, the nt-Cu/SiO_2_ hybrid bonds possess very low resistance and very low specific contact resistance. 

## 2. Materials and Methods

**Figure 1:**
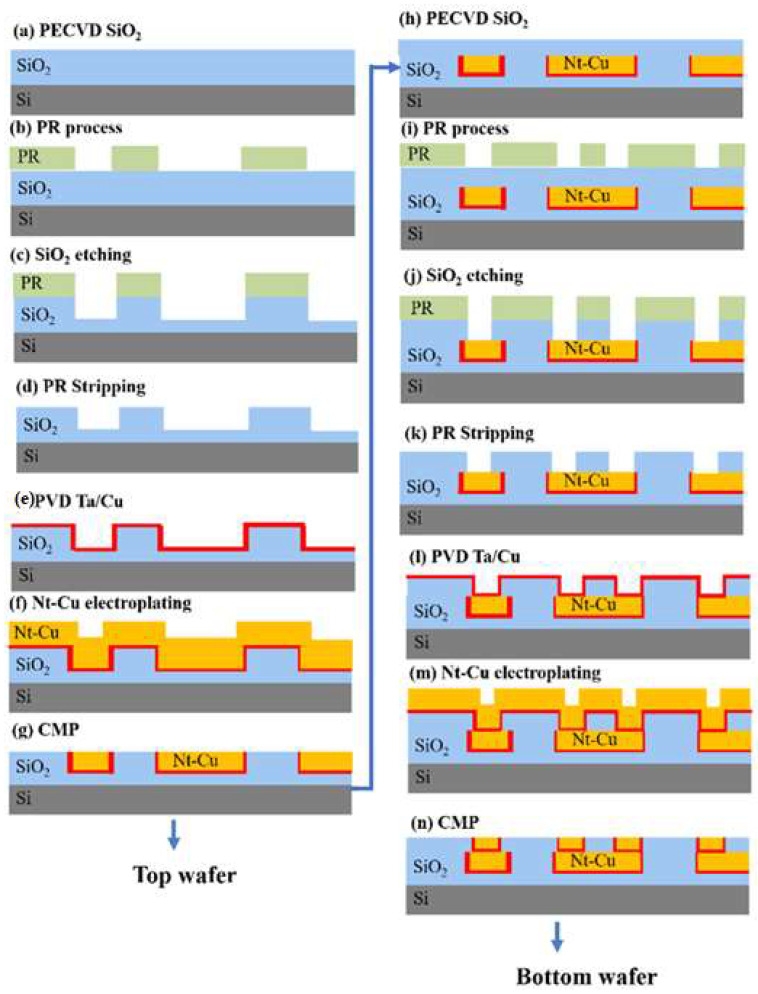
Schematic fabrication processes for top and bottom wafers.

The bonding schemes are 12-inch wafer-to-wafer. Initially, we fabricated 20 μm pitch Cu bumps with Cu redistribution layer (RDL) on top wafers and Cu RDL/microbumps on bottom wafers. Figure 1a–g displays the schematic processes for preparing the top wafers. A SiO_2_ film with 2 μm in thickness was initially deposited by plasma-enhanced chemical vapor deposition (PECVD, AMAT Producer SE, Applied Materials, Santa Clara, CA, USA), followed by lithography(SUSS Aligner MA300, SUSS MicroTec SE, Garching, Germany) and etching to define patterns. A Ta/Cu seed layer was deposited by physical vapor deposition. Highly (111)-oriented nt-Cu with 2 μm thickness was electroplated with bottom wafer fabrication, additional Cu vias measuring 8 μm in diameter were fabricated on the Cu RDL. Finally, by using chemical mechanical planarization (CMP, AMAT Reflexion LK Applied Materials, Santa Clara, CA, USA), the co-planarized nt-Cu/SiO_2_ surfaces were obtained, as depicted in Figure 1h–n. 

To achieve excellent recess control on the nt-Cu and SiO_2_, CMP was used to remove excess Cu and to control dishing on the bonding interface by a two-step process [17]. Proper dishing is desired for Cu/SiO_2_ hybrid bonding because the coefficient of thermal expansion for Cu is higher than that of SiO_2_. We will explain the details of the bonding mechanism later. With the two-step CMP process, the dishing performance can be well controlled within a few nanometers.

**Figure 2:**
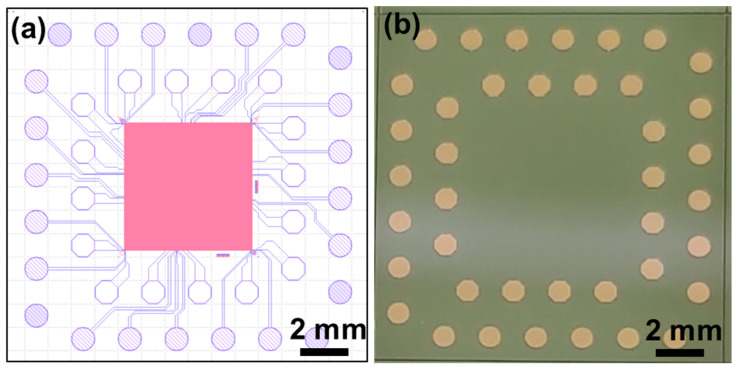
(**a**) Layout design for the test vehicles with top die bonded to bottom die. (**b**) Photo of a diced die.

For wafer-to-wafer hybrid bonding process, the as-prepared top and bottom wafers were cleaned by N_2_ plasma, followed by exposed in vacuum ambient (~10^−3^ mbar) under a contact force of 75 kN which is corresponding to 1.06 MPa and held at room temperature for pre-bonding, and the bonded pair was bonded at 200 °C for 1 h. The elevated temperature and pressure were further induced to the Cu surface to contact and begin diffusion bonding at the interface. To strengthen the bonding pair, post-annealing at 200 °C for 4 h was conducted, which may further improve both bonding strength and electrical properties. After the bonding process by a wafer level bonder (SUSS XBC-300, SUSS MicroTec SE, Garching, Germany) which the alignment accuracy can be controlled within ±1 μm, wafer grinding was conducted to thin down the top Si wafer to 20 μm. To expose the probing Cu pad, additional lithography and etching processes were conducted on the top wafer to open the probing pads. A dicing process was performed to slice 15 × 15 mm^2^ specimens for electrical and reliability tests. Figure 2a shows the designed layout for the test vehicle measuring 6 × 6 mm^2^ for the top die and 15 × 15 mm^2^ for the bottom die. There are 8000 Cu-Cu joints in each die. Figure 2b shows the optical photo for the fabricated die pair after the etching of the top die and the Cu probing pads exposed for electrical measurement.

For microstructure analysis, electron back-scattered diffraction (EBSD, JEOL JSM-7800F, Tokyo, Japan) was used to analyze grain size and crystal orientation. Microstructures and bonding quality were characterized by a focused ion beam (FIB, FEI Nova 2000, Hillsboro, OR, USA). Additionally, an atomic force microscope (AFM, Bruker Innova SPM, Billerica, MA, USA) was employed to obtain the random roughness patterns and determine the surface roughness (*R*_q_) of the nt-Cu vias. Confocal scanning acoustic microscopy (CSAM, Nordson SONOSCAN-Gen6, Elk Village, IL, USA) was used to examine voids non-destructively. For electrical resistance measurement, a source meter (Keithley 2700, Keithley, Instruments Cleveland, OH, USA) was adopted to measure the resistance of the Cu joints from −0.5 A to 0.5 A, and from room temperature to 375 °C. Kelvin probes were employed for the resistance measurement of a single Cu-Cu joint.

## 3. Results

### 3.1. Microstructural Characterization 

**Figure 3:**
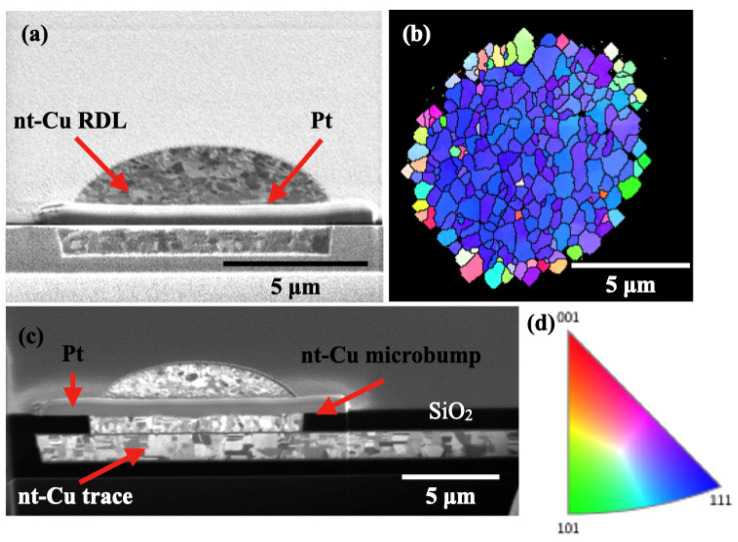
Microstructural characterization on the Cu via and joints before bonding. (**a**) Cross-sectional FIB image of unbonded nt-Cu RDL in the top die. (**b**) Plan-view EBSD analysis for a typical Cu bump on the test vehicle shows 78% of the via surface is (111)-preferred grains. (**c**) Cross-sectional FIB for the nt-Cu microbump on the nt-Cu trace in the bottom die. (**d**) Inverse pole figure of Cu.

The microstructures of the Cu pads after the CMP process were analyzed by FIB and EBSD. Figure 3a shows the cross-sectional FIB image of nt-Cu RDL on the top die before bonding. No obvious dishing was observed. The Pt layer was deposited to avoid damaging the Cu in the FIB during ion cutting. The plan-view EBSD shows 78% of the via surface is (111)-oriented grains, as shown in Figure 3b,c depicts the cross-sectional FIB for the nt-Cu microbump on the nt-Cu trace in the bottom die. The inverse pole figure is shown in Figure 3d. The nt-Cu trace was designed for the measurement of electrical resistance of the Cu-Cu joint.

**Figure 4:**
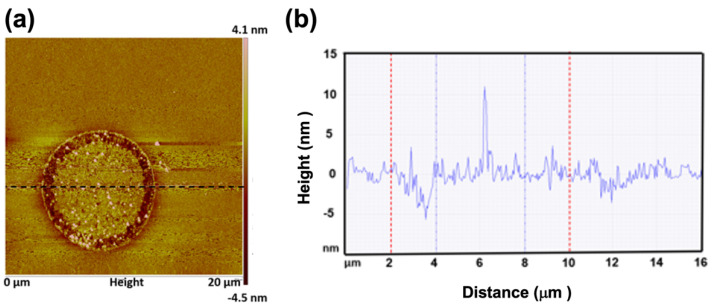
(**a**) AFM topography and (**b**) the results of analyzed surface roughness show that the recess of a typical Cu bump is less than 3 nm and Rq is less than 2 nm.

To facilitate hybrid bonding, the surface roughness of Cu and SiO_2_ need to be reduced by CMP to a few nanometers. Figure 4 shows the typical AFM results for a Cu via surrounded by SiO_2_ dielectrics. The root mean square averages of the individual heights and depths from the mean line (Rq) were measured for the Cu and SiO_2_ surface as 1.65 and 0.5 nm in average of six samples each, respectively. It is worth noting that the nt-Cu has a high hardness of 2.2 GPa, which minimizes the dishing after a CMP process. As shown in Figure 3a and Figure 4, the dishing/recess is quite uniform and can be controlled within 3 nm, which is good for bonding with a better interface geometry. 

It is reported that the Cu surface diffusivity on (111) planes is approximately 3–4 orders faster than that on (100) or (110) planes [13]. With ultra-high surface diffusivity, excellent Cu-Cu bonds can be achieved at 200 °C by wafer-to-wafer bonding. Such a bonding process with different adjustments of parameters was demonstrated to provide excellent mechanical properties [18,19,20,21,22] and high resistance in electromigration [18,23]. 

**Figure 5:**
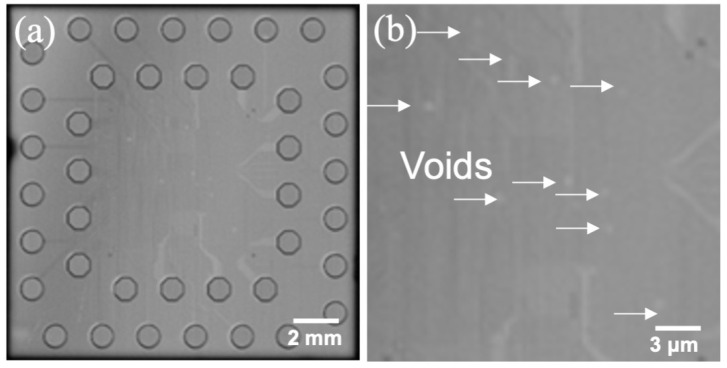
(**a**) CSAM results for bonded dies showing more than 95% areas are well bonded. (**b**) Enlarged image of bonded area with small voids indicated by pointed arrows.

Confocal scanning acoustic microscopy (CSAM) was used to examine bonding quality non-destructively. The resolution we adopted is good enough to observe the unbonded area in a die. Figure 5a shows the CSAM image for a bonded die pair and the results show that more than 95% of the bonding area was well bonded. We have also enlarged the CSAM analyzed image and found that just a few small voids were observed as shown in Figure 5b. 

**Figure 6:**
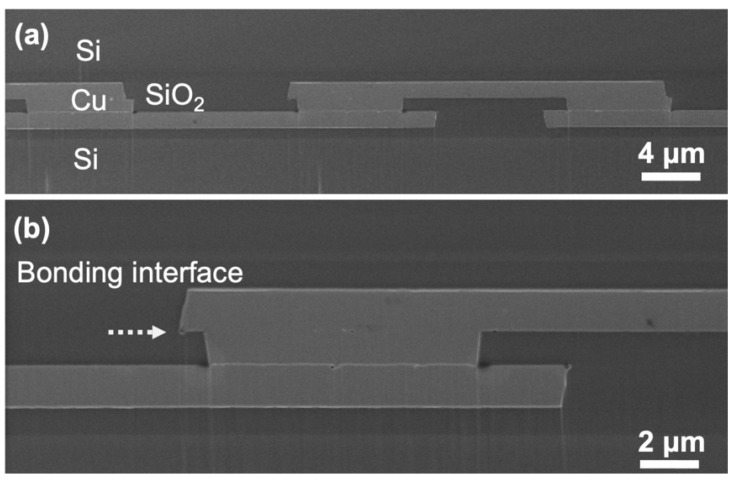
(**a**) SEM and (**b**) enlarged SEM images showing a row of Cu-Cu joints surrounded by SiO_2_ dielectrics.

Then the specimens were ion-milled by FIB for further bonding interface examination. Figure 6a shows cross-sectional SEM, respectively. Figure 6b presents an enlarged SEM image for a single Cu-Cu joint, which shows the Cu-Cu and SiO_2_-SiO_2_ are well bonded at this condition without obvious voids or cracks. 

### 3.2. Electrical Resistance Measured by Kelvin Probes

**Figure 7:**
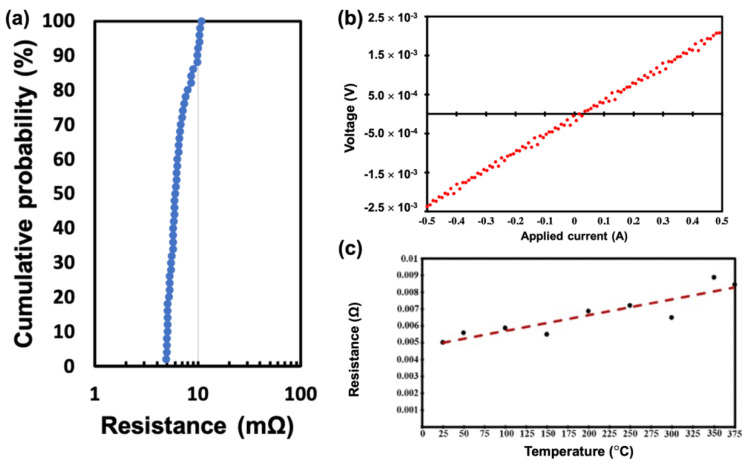
(**a**) Measured cumulative resistance for single Cu-Cu joint by four-point probes; (**b**) I–V curves; (**c**) Resistance against measured temperatures from 25 °C to 375 °C.

The nt-Cu/SiO_2_ hybrid bonds possess excellent electrical properties, although they were bonded at a low temperature of 200 °C. Electrical resistance was measured by four-point probes after the post-annealing process. Figure 7a shows the measured resistance for a Kelvin structure by wafer-to-wafer hybrid bonding approach. The total number of measured contact structures is 50 and the average resistance is 6.7 mΩ, while small variations in resistance of ±1.75 mΩ were observed. Similarly, crossbar modified Kelvin structures were also fabricated by Cu-Cu bonding, thus the bonding area can be fixed at 10 μm × 8 μm and the problem of misalignment can be ignored. The average resistance of 50 joints is 1.5 mΩ, which corresponds to a specific contact resistance of 1.2 × 10^−9^ Ω·cm^2^. So far, this value is the lowest among all the literature values for Cu-Cu joints bonded below 300 °C. Table 1 summarizes the corresponding published values with similar contact areas but at various temperatures. Usually, in order to achieve low specific contact resistance, the temperature for the bonding has to exceed 250 °C. By using the highly (111)-oriented Cu surface, low temperature bonding with low specific contact resistance can be achieved due to the high surface diffusivity and low oxidation rate. 

The hybrid joints also exhibit excellent thermal stability. Figure 7b presents the linear I–V curves using currents from −0.5 A to 0.5 A, and Figure 7c shows the resistance as a function of measured temperature up to 375 °C. These results show excellent thermal stability within the hybrid bonds.

### 3.3. Mechanism for Cu/SiO_2_ Hybrid Bonding

**Figure 8:**
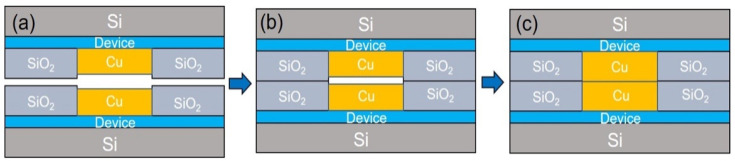
Schematic of the ideal bonding mechanism of the Cu/SiO_2_ hybrid system. (**a**) Sides with Cu bumps in SiO_2_ via. The height of the Cu bump is slightly lower than the surrounding SiO_2_ layer. (**b**) Alignment and bonding of SiO_2_ to SiO_2_ at room temperature. (**c**) Heating to close the dishing gap and induce pressure in the Cu bump. The pressure was created due to the large CTE of the Cu. No external pressure is needed at the third stage.

The mechanism for the Cu/SiO_2_ hybrid bonding is described below. The key step for the hybrid bonding is to control the height and shape of the Cu recess precisely. Figure 8 illustrates the schematic diagram for the hybrid bonding mechanism. After the CMP process, the height of the Cu via should be slightly lower than the surrounding SiO_2_ film, as shown in Figure 8a. The top and the bottom Si wafers are aligned face-to-face and compressed at room temperature to bond the top and the bottom SiO_2_ films first, as depicted in Figure 8b. Then, the bond wafers are heated to a suitable temperature without external pressure to further strengthen the SiO_2_ and SiO_2_ bonding, as well as to accomplish the Cu-Cu bonding, as seen in Figure 8c. The compressive stress needed to trigger the Cu-Cu bonding is generated internally by the difference in CTE mismatch between the PECVD SiO_2_ and Cu. It is reported that the PECVD SiO_2_ possesses a CTE value of 4.4 × 10^−6^/°C [30]; while it is 16.6 × 10^−6^/°C for Cu. During the heating process, the Cu expands more than the SiO_2_ does. Therefore, the upper and the lower Cu vias contact each other and compressive stress is created in the Cu vias. Then creep takes place in the contact interface of the Cu vias to achieve the Cu-Cu bonding [3,14]. 

However, on the other hand, the SiO_2_ layers are under tensile stress during the high temperature bonding process. When the Cu recess is too small, the expanding of Cu may apply high tensile stress in the oxide layer, causing a rupture of the SiO_2_ layers. When the recess of the Cu via is too much, the two Cu vias may not be able to contact each other and the bonding of Cu-Cu could not be accomplished. Therefore, the control of the Cu recess is critical in Cu/SiO_2_ hybrid bonding. The optimal height difference depends on the bonding temperature. The higher the bonding temperature is, the larger the height difference is. 

Furthermore, low temperature Cu/SiO_2_ possesses the following advantages. Some devices, such as high bandwidth memories, prefer low temperature (<230 °C) hybrid bonding to prevent data retention errors. In addition, wafer warpage and thermal stress is lower at a low temperature, and delamination between the SiO_2_ layers and other film structures in the active device region could be avoided. Nevertheless, low temperature bonding is very challenging. Because the Cu will expand less at low temperatures. Therefore, the optimal height difference is smaller, which means recess control on the Cu vias is more difficult. 

The maximum Cu recess could be estimated at a given bonding temperature. Typically, the height difference caused by CTE difference can be expressed as

ΔL = Δα L_0_ ΔT (1)

where *L_0_* is the thickness at room temperature in this study, Δ*L* is the height difference due to the mismatch between the thermal expansion of Cu and the oxide, Δ*α* is the difference in CTE, and Δ*T* is temperature difference. In this work, we take the Cu/oxide layer to be 2 μm thick, and the bonding temperature is 200 °C. The estimated maximum recess for the Cu via is 4.3 nm. It means that when the Cu dishing is larger than 4.3 nm, the two Cu vias would not be able to touch each other when the temperature is increased to 200 °C. In this work, we control the Cu recess height to be 3 nm. Therefore, the Cu vias expand approximately 4.3 nm at 200 °C to contact with each other and provide suitable compressive stress for the Cu-Cu bonding.

ΔL = Δα L_0_ ΔT = (16.6 − 4.4) × 10^−6^ × 2000 nm × (200 − 25) = 4.3 nm (2)

**Figure 9:**
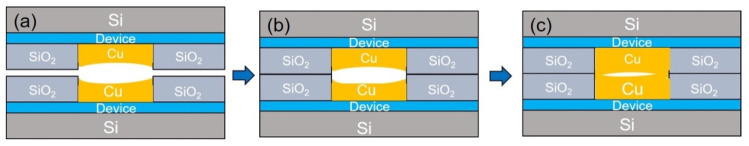
Schematic of the bonding mechanism with non-uniform bonding surfaces in a Cu/SiO_2_ hybrid system. (**a**) Sides with Cu bumps in SiO_2_ via. The non-uniformity (dish shape) on Cu surface is generated after the CMP process (**b**) Alignment and bonding of SiO_2_ to SiO_2_ at room temperature with dishing Cu. (**c**) The periphery of the Cu vias can be well-bonded after high temperature annealing. No external pressure is needed at the third stage.

It is also crucial to produce a uniform Cu recess for good bonding. Otherwise, large voids may occur to weaken the mechanical, electrical, and thermal performance. As schematically shown in Figure 9a,b, when non-uniformity (dish shape) is generated after the CMP process and after the bonding of the oxide layers, only the periphery of the Cu vias can be well-bonded after the high temperature annealing (Figure 9c). Large voids or seams may remain after the bonding process. The voids may not be able to be eliminated, even after a long annealing time. For nt-Cu vias, uniform recesses were obtained due to their high hardness of 2.2 GPa, as illustrated in Figure 3a and Figure 4. Therefore, there are no large voids observed in the bonding interface, and thus the specific contact resistance is low. 

## 4. Conclusions

By adopting the highly <111>-oriented nt-Cu, we can fabricate nt-Cu/ SiO_2_ hybrid bonds at 200 °C with a very low specific contact resistance of 1.2 × 10^−9^ Ω·cm^2^, which is the lowest value reported in literature for Cu-Cu bonds fabricated below 200 °C. Excellent thermal and electrical stability was obtained up to 375 °C. The main reasons for the excellent electrical performance are that the <111>-oriented nt-Cu possesses high surface diffusivity and low oxidation rate, thus the nt-Cu vias can be bonded at a low temperature. More importantly, the nt-Cu possesses high hardness and thus the height and shape of the Cu recess can be well controlled by CMP to realize the low temperature Cu/SiO_2_ hybrid bonding. The mechanism of hybrid bonding is also discussed, and a mismatch in thermal expansion of Cu and SiO_2_ was utilized to produce compressive stress for the Cu-Cu bonding. The results indicate that the nt-Cu is suitable for low temperature Cu/SiO_2_ hybrid bonding.

## Figures and Tables

**Table 1:** List of measured specific contact resistances from literature.

	Ref. [24]	Ref. [25]	Ref. [26]	Ref. [27]	Ref. [28]	Ref. [29]	This Work
Spec. Cont. R. (10^−8^ Ω.cm^2^)	0.12	0.505	0.282	0.30	2.6	0.15	0.12
Bonding Temp. (°C)	400	400	350	250	250	200	200
Contact Area (μm^2^)	100	100	80	80	32.5	9	80

## Data Availability

The data presented in this study are available on request from the corresponding author.
